# Seasonal Pattern of Advertisement Calling and Physiology in Prolonged Breeding Anurans, Japanese Tree Frog (*Dryophytes japonicus*)

**DOI:** 10.3390/ani13101612

**Published:** 2023-05-11

**Authors:** Jun-Kyu Park, Yuno Do

**Affiliations:** Department of Biological Science, Kongju National University, Gongju 32588, Republic of Korea

**Keywords:** breeding timing, frog chorus, physiology, anurans, prolonged breeder species

## Abstract

**Simple Summary:**

In this study, we attempted to identify the seasonal pattern of reproductive emergence in the Japanese tree frog (*Dryophytes japonicus*). Individuals that emerged early in the breeding season tended to have relatively high energy storage and conserved energy used for advertisement calling. In the middle of the breeding season, the chorus size of male frogs was the highest, indicating a breeding peak. Individuals that emerged at this time showed signs of energy depletion and low immunity. Towards the end of the breeding season, male frogs had high energy storage and immunity, likely due to the influx of new individuals at this time. During this period, individuals displayed rapid cries for breeding spurts. Our findings imply that prolonged breeder species may adjust their breeding participation in response to the risks and benefits of breeding timing.

**Abstract:**

The calling behavior of anurans should be studied in detail as it greatly influences their physiology and immunity, particularly in prolonged breeding species. The effect can be further complicated by the emergence timing in the breeding season. We conducted a study comparing the physiology and calling behavior of the Japanese tree frog (*Dryophytes japonicus*), a prolonged breeder species, according to the breeding timing. During the middle of the breeding season, a high chorus size appeared, indicating a breeding peak. However, chorus size did not dominate physiology and calling behavior. In the early breeding season, frogs had a high energy storage state and immunity. In the middle of the breeding season, individuals from the early breeding season were considered to have exhausted their energy stores and had low immunity. Towards the end of the breeding season, frogs appeared to have newly introduced, at which time energy stores and immunity were as high as in the beginning. However, unlike the physiology, the pattern of calling constantly varied as the breeding season progressed. Frogs from the early season conserved energy used for calling, and frogs from the late season showed a breeding spurt for mating. Our results can help in understanding the energy metabolism of calling behavior, physiology, and disease epidemiology in prolonged breeder species. They also suggest that individuals coordinate their participation in the breeding season and that the timing of their appearance at breeding sites may not be random.

## 1. Introduction

Anurans generate advertisement calls for reproduction, which are considered some of the most energetically demanding aerobic activities among vertebrate taxa [1]. Male anurans spend most of the breeding season engaged in calling activity, and many factors in their life history are affected by calling, such as mortality, reproductive success, energy metabolism, and immune regulation. During advertisement calling, male frogs use stored fat or gain energy from feeding activities that parallel calling [2,3]. Male frogs may participate in a chorus with other males, creating calls together instead of calling individually. This can cause competition for calling and the attraction of males, but it can reduce the rate of energy expenditure per hour, allowing longer stays in the breeding sites. Additionally, high density of chorus size may increase the mating chances for male frogs by increasing female participation rates [4]. Some species conserve energy by alternating calls at high chorus densities or exhibiting periodic silences concurrently with each other. However, they also increase calling effort and energy expenditure when competition is intense [5,6]. Increased calling efforts may attract more females [7] but may also reduce body mass or immunity due to increased energy expenditure [8,9]. Immunity may interact with steroid hormones such as testosterone, which governs male estrus and determines the length of reproduction, or corticosterone, which can regulate glucose metabolism to elicit an energy response or to respond to stress [10,11].

Further study is needed to understand the regulation of calling behavior, physiology, and immunity, depending on the breeding strategy of anurans. Breeding strategies can be broadly divided into two types: exclusive breeder species and prolonged breeder species, based on the intensity and period of breeding [12,13,14]. Exclusive breeder species form dense assemblages with large spatial and temporal overlap, and females arrive at the breeding sites simultaneously. Males engage in scramble competitions, which involve physical competition [15,16]. In contrast, prolonged breeder species have asynchronous female arrival at breeding sites, and males maintain individual sites for a period of time to generate advertising calls [17]. Prolonged breeder species remain at breeding sites for several months, with spawning occurring periodically to maintain a continuous supply of offspring. More research and understanding of prolonged breeder species are needed as they can promote or delay reproduction depending on the changing environments in breeding sites. In particular, males of prolonged breeder species may be at high risk of long-term energy consumption as they do not know when the female will arrive and must continue calling until their energy reserves are exhausted [18]. Nonetheless, the longer they call, the greater the chance of mating success [19], so they remain there until mating. Eventually, they can make choices about when to engage in breeding during the long breeding season. If frogs cannot independently choose when to appear, environmental factors at the breeding sites or physiological changes that control estrus may be important. It may depend on the attractiveness or energy stores of the frogs. To understand the advantages and risks of breeding strategies that prolonged breeder species have over explosive breeder species, it is necessary to identify the temporal pattern by identifying their body conditions and calling variation by the time of their emergence. Additionally, since the appearance or maintenance patterns of breeding sites may differ depending on the species, research on this is necessary.

Before the chorus of frogs is created, male frogs in the early stages of breeding take risks in exploring the breeding grounds. They can expend energy on the search and, in the process, may become isolated, dehydrated, or face the risk of predation [20]. Early breeding carries risks, but it also has many benefits. It may help the tadpoles they bred compete for resources and may increase fertilization success. Additionally, male frogs can quickly reach an active period in their life history when they can reduce energy consumption for reproduction and accumulate energy. On the other hand, later breeding may reduce the energy used for searching for breeding sites but may result in a smaller population of females participating in breeding and lower chances of mating. Males emerging in the middle of the breeding season may have intermediate advantages and disadvantages in both of these but may experience strong competition due to the peak of the breeding season. This not only reduces the success of mating but can also affect disease dynamics or stress [15,21]. They alternatively conserve the energy they use by calling together, reducing their energy consumption per hour, and allowing them to remain in the breeding ground for a longer period of time. Even within the breeding season, there are risks and advantages that appear at each timing, and the attributes of male frogs that appear at this time may be different.

In this study, we aimed to identify differences in individual attributes of male tree frogs (*Dryophytes japonicus*), during the breeding period. This prolonged breeder species of frog is a common species in East Asia, including China, Japan, and Korea [22] and is a species that appears in breeding grounds from spring to summer [2,23]. We wanted to test the assumptions that there is only one breeding peak in the middle of the breeding season, the population density is at its peak at this time, and that the physiological state and calling pattern of the individuals during this period gradually change. Males of prolonged breeder species call for a longer period of time before mating, resulting in more high-energy activity. Accordingly, the state of energy storage is important to them [3]. Reproductive behavior in male frogs is often triggered by steroid hormones such as testosterone and corticosterone [24,25]. Specifically, the excessive investment of energy used for calling in the breeding season may result in a reduction in immune function, potentially due to the energetic demands of sustained reproduction and the trade-off between allocation of resources to reproduction versus immune defense [9]. This immune regulation may occur between energy balance and sex hormones [26], and further research is needed as reduced immunity may make frogs more susceptible to infection and disease. We attempted to confirm this using the percentage of fat to represent the energy storage state and the bacterial killing ability in the blood, which can be used to measure the immune state.

## 2. Materials and Methods

### 2.1. Field Survey

*D. japonicus* is a prolonged breeding species that generally breeds from spring to summer in Korea. We selected three sites with paddy fields in Gongju-city where we could study this species from spring to summer 2022. Three teams, each consisting of two experts, conducted simultaneous field investigations at each location. Breeding initiation for frogs at the three locations began in late April. From this time, we started recording frog calls and collecting frogs. We designated this point as the 1st round of field investigation. Five frogs per location were used for analysis, resulting in a total of 15 frogs sampled per round. At the beginning of the experiment, temperature and humidity were measured three times at each location. The final field investigation was completed in late June. We were able to conduct a total of five samplings at 2-week intervals from the start date. After the fifth round of field investigation, a few frogs were calling for a week, but there was no calling when we tried to sample 2 weeks later. Therefore, we designated the fifth round of field investigation as the last sampling. A total of 75 frogs were used for the analysis of calling traits, chorus size, hormones, immunity, and physiological condition from five rounds. Experimental procedures involving animals were performed in accordance with the regulations and approval of the Experimental Animal Ethics Committee of Kongju National University (KNU_2022-01).

### 2.2. Calling Analysis

We recorded the calling of the frogs using the same method as previously studied [8]. Frog calls were recorded on site. We carefully approached the frogs and recorded their calls for 5 min using a shotgun microphone (ZOOM F1-SP, Tokyo, Japan) at a distance of at least 1 m from each frog. If any individuals escaped from the calling site before 5 min of recording time or could not be collected after recording, new individuals were recorded without being included in the experiment. To avoid recording calls or collecting individuals more than once, the experiment was conducted by moving only in a straight line. Additionally, the chorus size was determined based on the individuals recorded in the field. In each two-person team, one person targeted the individuals and recorded their calls, while the other measured the chorus size. The chorus size was calculated by counting the number of other frogs calling within a 5 m × 5 m area from the point where an individual’s call was recorded.

**Figure 1:**
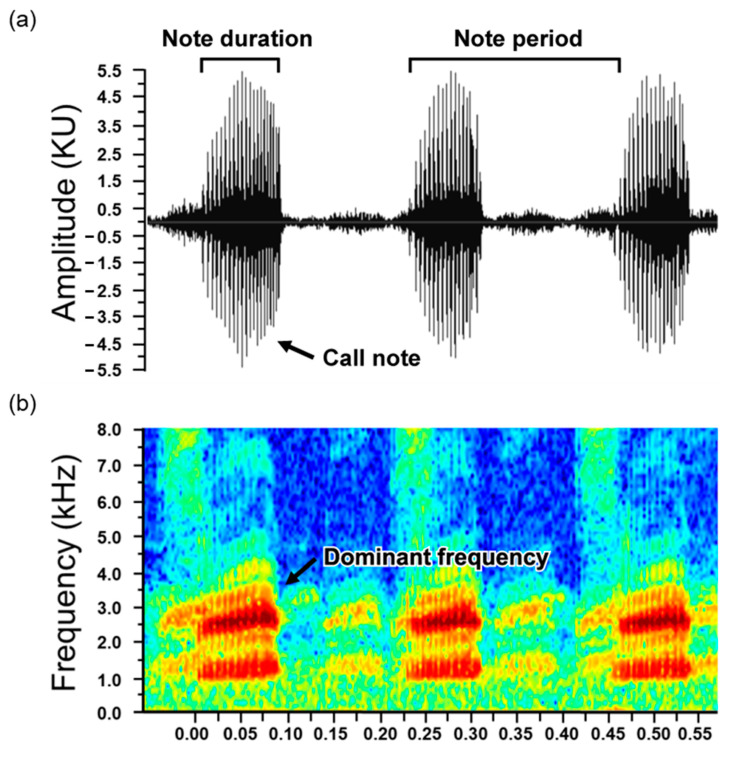
The oscillogram and sound spectrogram of a Japanese tree frog’s call. Panel (**a**) displays the call note and duration, as well as the note period used for call rate calculation. Panel (**b**) illustrates the dominant frequency of the call in the sound spectrogram.

The calling traits were analyzed using Raven Pro software version 1.6 (Cornell Laboratory of Ornithology, Ithaca, NY, USA). We analyzed three traits of frog calls (Figure 1): (1) note duration, indicating the length of the note, calculated by subtracting the start time from the end time of the note; (2) dominant frequency, representing the frequency with the most energy; and (3) call rate, calculated by the formula (1/note period (note-to-note spacing: the interval between the start time of a note and the start time of the next note in continuous calling)), indicating the speed of the call. We analyzed 20 call notes per individual, and the average value was used as a representative of the call traits for each individual.

### 2.3. Blood Extraction

After recording the frog calls in the field, the individuals were collected. All collected frogs were transported to the laboratory within 2 h. Frogs were anesthetized with ice-cold water, and blood was drawn through cardiac venipuncture using a syringe. Blood extraction was also carried out in the same method as previously studied [8]. Since the blood volume was very low, the blood was transferred from the syringe to a heparinized capillary tube and then to a 1.5 mL microcentrifuge tube using a pipette. The blood was centrifuged at 3000× *g* for 5 min, and the supernatant was extracted to obtain plasma. The extracted plasma was used to evaluate plasma testosterone and bacterial killing ability (BKA), which is one of the indicators of innate immunity in ecological immunology. After blood extraction, the snout-vent length, an indicator of anuran body size, was measured using a digital caliper to the nearest 0.01 mm. Additionally, body weight was measured using a digital balance to the nearest 0.01 g. Finally, the frogs were euthanized under anesthesia for further analysis.

### 2.4. Hormonal and Immune Assay

Steroid hormone was extracted from plasma by referring to a previous study [27]. Specifically, 3 mL of ethyl ether was added to 10 µL of the plasma sample, followed by vortexing, and centrifugation at 218× *g* at 4 °C for 9 min to separate the ether layer. Subsequently, plasma samples were rapidly frozen at −80 °C and held for 7 min. Then, the ether supernatant containing the free hormone was decanted into a new tube and stored in a laminar flow hood at room temperature (23 ± 1 °C) to completely dry the ether. Finally, it was resuspended using the ELISA buffer provided by the 96-well testosterone ELISA kit (582701, Cayman Chemical, Ann Arbor, MI, USA).

Plasma testosterone concentrations were assayed according to the manufacturer’s instructions using a 96-well testosterone ELISA kit (582701, Cayman Chemical, Ann Arbor, MI, USA). Three different hormone plates were developed repeatedly at the same time, and the average value of the three plate measurements was used as a representative value for the individual. A microplate spectrophotometer (wavelength 412 nm) was used to read the absorbance of the plate. The inter-assay coefficient of variation (CV) based on each sample was <13.8%, which was suitable for use in the analysis.

A bacterial killing assay was performed using the extracted plasma according to the protocol of a previous study [28]. First, the plasma sample was diluted 1:20 using amphibian Ringer’s solution (total volume 200 µL), and then 10 µL of non-pathogenic Escherichia coli (Microbio-Logics #24311-ATCC 8739, St. Cloud, MN, USA) working solution (approximately 10^4^ microorganisms) was added. We used 210 µL of amphibian Ringer’s solution as a negative control, and a solution obtained by mixing 200 µL of Ringer’s solution with 10 µL of *E. coli* working solution as a positive control. All controls and samples were incubated at 37 °C for 60 min, then 500 µL of tryptic soy broth was added. The solutions were thoroughly mixed, and the suspensions were transferred to 96-well plates in duplicate in 300 µL aliquots. The plate was incubated at 37 °C for 2 h and measured at 1 h intervals on a microplate spectrophotometer (wavelength 600 nm) for a total of six times. The BKA was evaluated at the beginning of the bacterial exponential growth phase using the formula ((1) − (optical density of sample/optical density of positive control)), which represents the percentage of killed microorganisms in the plasma samples compared to the positive control.

### 2.5. Body Composition Measurements

Body composition, such as fat mass and muscle mass, can indicate an individual’s health, nutrition, or metabolic condition [29]. We utilized dual-energy X-ray absorptiometry (DEXA) with the Medikors InAlyzer (Seongnam, Korea), which alternates between transmitting high and low dose X-rays, to calculate the fat content and lean body content of the frogs. Lean body content is generally considered in DEXA as the sum of water content and muscle content, and this equipment follows the same principle [30,31]. Since we wanted to consider lean body content as muscle content, we kept the samples in 70% ethanol for 2 months to remove the maximum amount of body water before proceeding with DEXA measurements. All samples were kept in ethanol for the same period of time.

### 2.6. Statistical Analysis

Components such as body size, body length, and chorus size of frogs, along with temperature and humidity recorded in the field, were analyzed across five rounds using the Kruskal–Wallis test. Physiological conditions such as BKA, fat content, lean body content, and testosterone, as well as calling components (note duration, dominant frequency, and call rate), were also analyzed across five rounds through the Kruskal–Wallis test. Dunn’s post hoc test was performed when significance was found in the Kruskal–Wallis test. The Spearman correlation analysis was employed to identify correlations among chorus size, physical condition (body length and weight), physiology (BKA, fat content, lean body content, and testosterone), and call parameters (note duration, dominant frequency, and call rate).

Principal component analysis (PCA) with parallel analysis and oblique (promax) rotation was performed to cluster components with similar trends among chorus size, physical condition (body length and weight), physiology (BKA, fat content, lean body content, and testosterone), and call parameters (note duration, dominant frequency, and call rate). The oblique (promax) rotation was chosen because it assumes a correlation among each component. Factor loading was considered insufficient when less than 0.4, appropriate between 0.4 and 0.6, and high when greater than 0.6. Statistical analyses were performed using GraphPad Prism software (version 8.00, GraphPad Software, San Diego, CA, USA) and JASP software (version 0.16.4.0) [32]. All statistical results were considered significant at *p* < 0.05.

## 3. Results

### 3.1. Environmental Factors and Physical Conditions

From the first round of field investigation (R1) in late April to the fifth round of field investigation (R5) in late June, temperature (H = 65.53, *p* < 0.01) and humidity (H = 68.89, *p* < 0.01) increased steadily (Table 1).

**Figure 2:**
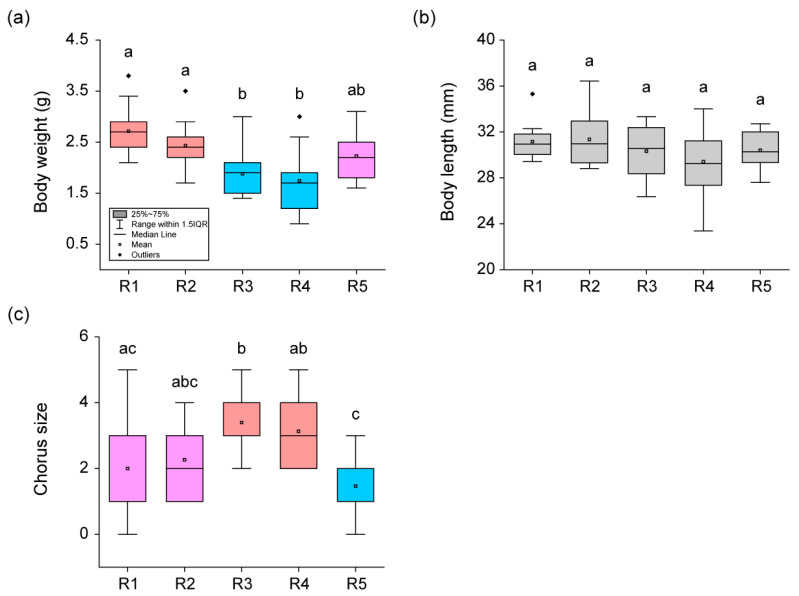
Comparison of body weight, body length, and chorus size among five observation rounds (R1–R5) during the breeding season. Panel (**a**) displays the differences in body weight among the five rounds. Panel (**b**) compares the body length of frogs from the five rounds. Panel (**c**) shows the change in chorus size among the five rounds. Significant differences (*p* < 0.05) were calculated using Dunn’s Post Hoc test following the Kruskal–Wallis test and are represented with lowercase letters. The red boxes indicate the collection round with the highest statistical significance, and the blue boxes represent the collection round with the lowest statistical significance. The pink boxes indicate the collection round of frogs with statistical significance between the red and blue boxes, while the gray boxes represent data without statistical significance. Box plots represent the mean values (central square dot), median values (central line), 25th and 75th percentile values (bottom and top of boxes), range within 1.5 interquartile (bottom and top of lines), and outliers (rhombus dots).

There was a significant difference (H = 29.19, *p* < 0.01) in body weight and chorus size (H = 23.33, *p* < 0.01) among the five rounds, whereas there was no difference (H = 5.22, *p* = 0.27) in body length (Figure 2). Body weight was the highest (Dunn’s Post Hoc, *p* < 0.05) in frogs from R1, gradually decreased, and was the lowest (Dunn’s Post Hoc, *p* < 0.05) in frogs from R4. In R5, the body weight of frogs increased and was not significantly different (Dunn’s Post Hoc, *p* > 0.05) from the body weight of frogs at the beginning of breeding. The chorus size remained consistent (Dunn’s Post Hoc, *p* > 0.05) in R1 and R2 but increased rapidly (Dunn’s Post Hoc, *p* < 0.05) in R3 and R4. Afterwards, in R5, the chorus size decreased rapidly (Dunn’s Post Hoc, *p* < 0.05) and it reached its lowest point.

### 3.2. Physiological Condition

**Figure 3:**
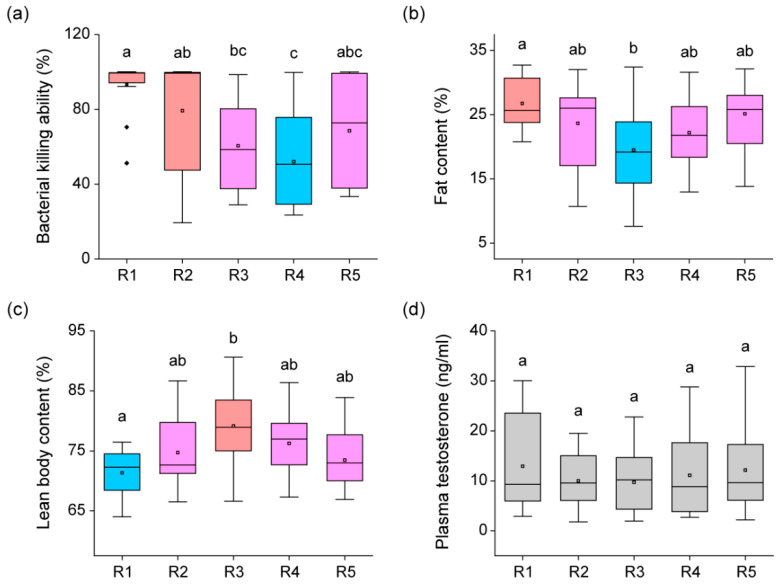
Comparison of physiological condition among five observation rounds during the breeding season. Panel (**a**) displays the difference in bacterial killing ability among frogs from the five rounds (R1–R5). Panel (**b**) shows the difference in fat content ratio of frogs from the five rounds. Panel (**c**) displays the difference in lean body content ratio of frogs from the five rounds. Panel (**d**) compares the plasma testosterone concentration in frogs from the five rounds. Significant differences (*p* < 0.05) were calculated using Dunn’s Post Hoc test following the Kruskal–Wallis test and are represented by lowercase letters. The red boxes represent the collection round of frogs with the highest statistical significance, and the blue boxes represent the collection round of frogs with the lowest statistical significance. The pink boxes indicate the collection round of frogs with statistical significance between the red and blue boxes, while the gray boxes indicate data without statistical significance. Box plots represent the mean values (central square dot), median values (central line), 25th and 75th percentile values (bottom and top of boxes), range within 1.5 interquartile (bottom and top of lines), and outliers (rhombus dots).

Bacterial killing ability (BKA) (H = 20.24, *p* < 0.01), fat content ratio (H = 12.71, *p* = 0.01), and lean body content ratio (H = 15.14, *p* < 0.01) showed significant differences, whereas plasma testosterone concentration did not significantly differ (H = 1.31, *p* = 0.86) among the five rounds (Figure 3). BKA was the highest (Dunn’s Post Hoc, *p* < 0.05) in R1 and R2, gradually decreased, and reached its lowest point (Dunn’s Post Hoc, *p* < 0.05) in R4. In R5, it became similar (Dunn’s Post Hoc, *p* > 0.05) to the BKA of frogs in the middle of the breeding season. Fat content was the highest (Dunn’s Post Hoc, *p* < 0.05) in R1, gradually decreased, and reached its lowest point (Dunn’s Post Hoc, *p* < 0.05) in R3. Thereafter, there was a slight increase in R4 and R5, with no significant difference (Dunn’s Post Hoc, *p* > 0.05). In contrast to fat content, lean body content was the lowest (Dunn’s Post Hoc, *p* < 0.05) in R1, gradually increased, and reached its highest point (Dunn’s Post Hoc, *p* < 0.05) in R3. Thereafter, there was a slight decrease in R4 and R5, with no significant difference (Dunn’s Post Hoc, *p* > 0.05).

### 3.3. Change of Calling Pattern

**Figure 4:**
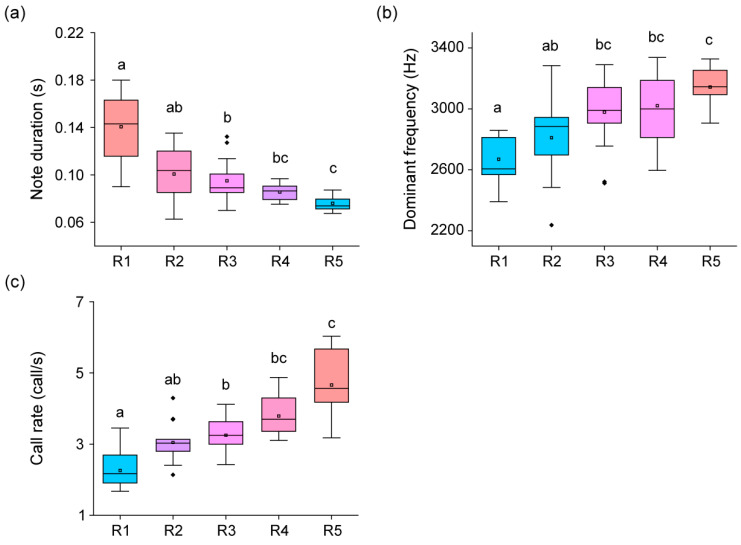
Comparison of calling patterns among five observation rounds during the breeding season. Panel (**a**) displays the difference in note duration among frogs from the five rounds (R1–R5). Panel (**b**) shows the difference in dominant frequency of frogs from the five rounds. Panel (**c**) displays the difference in call rate of frogs from the five rounds. Significant differences (*p* < 0.05) were calculated using Dunn’s Post Hoc test from Kruskal–Wallis test and are represented by lowercase letters. The red boxes represent the collecting round of frogs with the highest statistical significance, and the blue boxes represent the collecting round of frogs with the lowest statistical significance. The pink boxes indicate the collecting round of frogs connected with a statistical significance between the red and blue boxes. Box plots represent the mean values (central square dot), median values (central line), 25th and 75th percentile values (bottom and top of boxes), range within 1.5 interquartile (bottom and top of lines), and outliers (rhombus dots).

All call parameters, including note duration (H = 44.15, *p* < 0.01), dominant frequency (H = 33.38, *p* < 0.01), and call rate (H = 50.34, *p* < 0.01), had significant differences (Figure 4). Note duration was the highest (Dunn’s Post Hoc, *p* < 0.05) at R1, rapidly decreased, and reached its lowest point (Dunn’s Post Hoc, *p* < 0.05) at R5. The dominant frequency was the lowest (Dunn’s Post Hoc, *p* < 0.05) in R1 and R2, gradually increased, and reached its highest point (Dunn’s Post Hoc, *p* < 0.05) in R5. The call rate was exceptionally low (Dunn’s Post Hoc, *p* < 0.05) in R1, rapidly increased, and became exceptionally high (Dunn’s Post Hoc, *p* < 0.05) in R5.

### 3.4. Relationship of Call Parameters and Physiology Components

**Figure 5:**
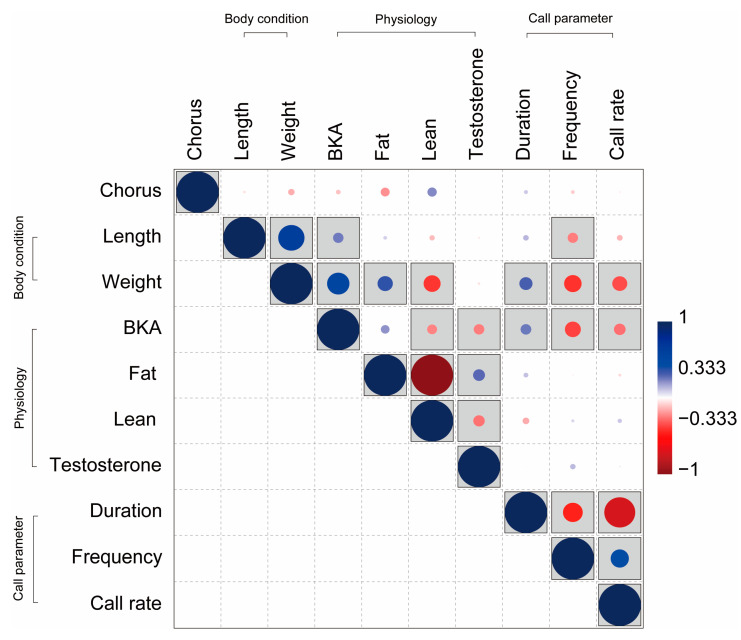
Correlations among chorus size, body condition (length and weight), physiological condition (bacterial killing ability (BKA), fat content, and lean body content), and call parameters (note duration, dominant frequency, and call rate) were analyzed using Spearman correlation analysis. The correlation plot displays the Spearman correlation coefficient, with the color gradient indicating the strength of the correlation. Significant correlations (*p* < 0.05) are represented by gray boxes.

In the Spearman correlation analysis, chorus size did not correlate (*p* > 0.05) with any of the variables. Some correlated components were identified in body conditions (body length and body weight), physiological conditions (BKA, fat, lean, and testosterone), and call parameters (note duration, dominant frequency, and call rate) (Figure 5).

The increase in body length correlated only with the increase in BKA (Spearman’s r = 0.246, *p* = 0.03) among physiological conditions, and only with the decrease in dominant frequency (Spearman’s r = −0.245, *p* = 0.03) among call parameters. Increased body weight also correlated with increased fat (Spearman’s r = 0.361, *p* < 0.01) and decreased lean (Spearman’s r = −0.402, *p* < 0.01), including increased BKA (Spearman’s r = 0.528, *p* < 0.01), and was correlated with all call components. An increase in body weight resulted in increased note duration (Spearman’s r = 0.310, *p* < 0.01) and decreased dominant frequency (Spearman’s r = −0.413, *p* < 0.01) and call rate (Spearman’s r = −0.355, *p* < 0.01).

Increased BKA was associated with decreases in lean body content (Spearman’s r = −0.234, *p* = 0.04) and testosterone (Spearman’s r = −0.250, *p* = 0.03), and was correlated with all call parameters. An increase in BKA, like body weight, resulted in increased note duration (Spearman’s r = 0.252, *p* = 0.03) and decreased dominant frequency (Spearman’s r = −0.376, *p* < 0.01) and call rate (Spearman’s r = −0.275, *p* = 0.02). Increased testosterone was only related to physiological conditions, such as increased fat content (Spearman’s r = 0.281, *p* = 0.01) and decreased lean body content (Spearman’s r = −0.270, *p* = 0.02). Among the call parameters, increasing note duration resulted in a decrease in dominant frequency (Spearman’s r = −0.466, *p* < 0.01) and call rate (Spearman’s r = −0.732, *p* < 0.01).

**Figure 6:**
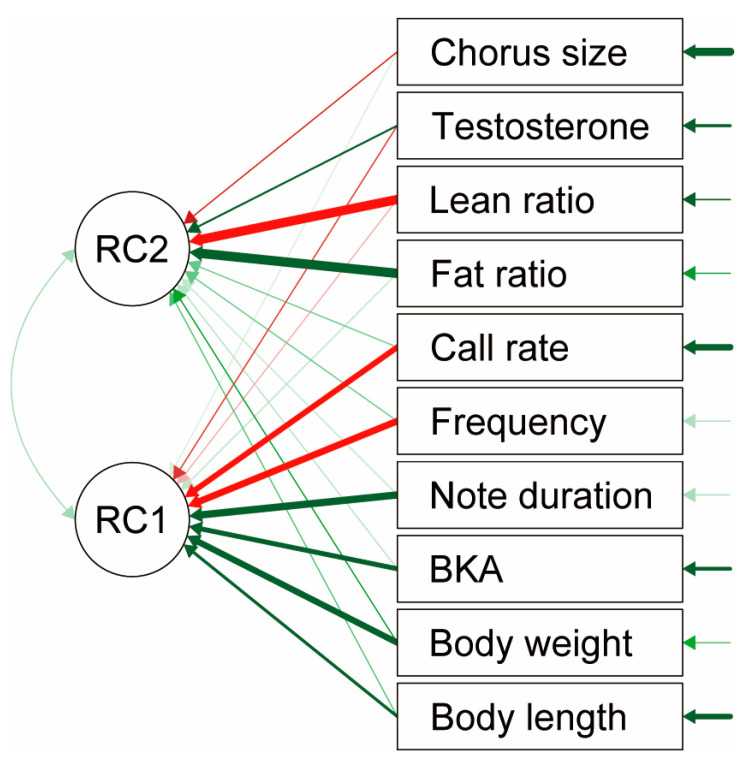
Path diagram showing the two major components of chorus size, body condition (length and weight), physiological condition (bacterial killing ability (BKA) fat content, and lean body content), and call parameters (note duration, dominant frequency, and call rate) obtained by principal component analysis (PCA). Negative correlations are represented by red lines, positive correlations by green lines, and the width of the lines indicates the strength of the correlation.

We also used PCA to cluster components with similar trends. A chi-squared test demonstrated an adequate model fit (χ^2^ = 240.66, *p* < 0.01). The 10 components consisting of chorus size, body conditions, physiological conditions, and call parameters were divided into two major components (Table 2, Figure 6). RC1 consists of three call parameters (note duration, dominant frequency, and call rate), body conditions (body weight and body length), and BKA, which represents immunity among physiological components. RC2 consists of fat content and lean body content representing body composition in physiological components, and testosterone representing sex hormone status in physiological components. Chorus size loading coefficients were lower than 0.4 for both RC1 and RC2, so they did not belong to either group.

## 4. Discussion

Here, we aimed to identify physiological and behavioral traits of Japanese tree frogs during their prolonged breeding season. During the early stages of the breeding season, male frogs may face several challenges, including expending significant amounts of energy to locate suitable breeding grounds, which can come with risks such as predation, desiccation, and isolation [20]. The lower chorus size early in the breeding season in our study suggests that this risk may be more extreme. It is not known how long male frogs of prolonged breeder species remain on the breeding sites, but they usually slowly leave the breeding sites once mating is complete. As a result of a study on *Hyla gratiosa*, a prolonged breeder species with a breeding period of up to 5 months, some males may remain for more than a month, but most of them stay for only 2 to 3 days, and about 75% of males stay for less than a week. This suggests that individuals within the population can be rapidly cycled [12]. Similarly, our results showed that the initial body weight, immunity, and fat content, which can be rapidly mobilized for call production, were high and decreased over time. However, this is not a result of individual tracking, and it was difficult to know whether individuals with reduced fat content and immunity in the middle of the breeding season were the individuals advertising calling in the initial breeding. In prolonged breeder species, male frogs have very good health in initial breeding and lose weight over time [18]. Our findings indicate that individuals with low fat content and immunity at the beginning of the breeding season are likely the same individuals present in the middle of the breeding season. As chorus density increases in the middle of the breeding season, individuals experience more extreme competition and consequently, energy consumption increases. This may result in lower immunity and fat mass. However, we ruled out this assumption as changes in dominant frequency, note duration, and call rate were inconsistent. On the other hand, individuals with high fat content and immunity in the late breeding season are likely to be newly introduced individuals. This suggests that individuals in *D. japonica* can be maintained for up to four weeks during the breeding season.

A high chorus size was observed during the middle of the breeding season, which may represent the peak of the breeding season for this species. We anticipated that almost all physiological and calling traits would vary during this peak. However, we found that chorus size was not related to body condition, physiology, or call parameters. There was no difference in body size at any time, but there was a large change in body weight, suggesting that this could be attributed to changes in fat content. In fact, our results show that fat and lean body mass ratios were more strongly correlated with weight than with body size [33,34]. This high-energy activity also decreases immunity [9]; a previous study showed that the enhanced call rate in Japanese tree frogs led to decreased immunity due to energy trade-offs [8]. Similarly, our results suggest that tree frogs have the lowest immunity during the middle of the breeding season, which may require attention to disease dynamics.

In general, lower dominant frequencies of calling are associated with larger body sizes [35], and females are known to prefer frogs with lower frequencies of calling [36,37]. Male frogs also have the ability to adjust their call rate in response to background noise or chorus size [8,38,39]. Our results showed that during the early breeding season, individuals exhibited a low dominant frequency and call rate, and a high note duration. This strategy may serve as an energy-saving mechanism during calling, as females might not have arrived at the breeding site yet. Despite the initially low chorus size, which could actually result in an increase in call rate, male frogs maintained a low call rate. A high call rate quickly drains energy, causing advertisement calls to end sooner during the day and during the breeding season [12]. Additionally, the difference in temperature between the early and late breeding seasons may also have an effect on call rate. Although temperature itself does not significantly affect the call rate, calling efficiency can certainly decrease at lower temperatures [1]. Since calling is performed at a low temperature during the early breeding season, the low call rate, even during a low chorus, appears to be an adaptive response. Furthermore, it is unclear whether this species can self-regulate the dominant frequency. Correlation analysis revealed that body size and weight could influence dominant frequency. This passage suggests that both physiological and physical factors can influence calling parameters, such as dominant frequency. Among the physiological parameters we measured, only BKA showed a direct relationship with calling parameters. However, since each physiological parameter is interconnected, it may indirectly impact other parameters. For instance, prior research discovered that testosterone supplementation did not generally increase immunity, but only in individuals with higher body mass [11]. This indicates that the immune-boosting effect of testosterone requires the support of body weight. In fact, weight is connected to body composition, which is related to testosterone, testosterone to BKA, and BKA to body size and weight. Therefore, it appears that these factors are indirectly linked and influence calling parameters. Consequently, variations in individual traits can alter calling characteristics, such as differences in dominant frequencies, during the breeding season. However, we still lack a precise understanding of how the interactions among numerous physiological variables affect vocalizations, so further research is necessary.

Unlike the early breeding season, the low chorus size was the same in the late breeding season, but the call rate increased rapidly. On the other hand, the fat ratio and immunity showed higher conditions. The newly introduced individuals in R4 and R5 likely recognized that they were late in the breeding season and demonstrated spurts in the late breeding season. This potentially suggests that frogs may be aware of conditions during the breeding season and adjust their participation accordingly. In fact, during the breeding season, some Japanese tree frogs feed while others participate in breeding [2]. This observation potentially suggests that frogs may be aware of their own condition or the conditions at the breeding sites, and that individuals may choose the appropriate timing to participate in breeding activities.

Another hypothesis is that individuals that appear early in the breeding season may differ in size, growth rate, and sexual maturity after metamorphosis from individuals that appear later in the breeding season. This may be restricted to only males participating in breeding for the first time (2nd or 3rd year old) but could ultimately determine when they engage in the breeding season. This could be very important for the adaptation and evolution of prolonged breeder species, and since we still lack detailed knowledge, further research is necessary.

## 5. Conclusions

**Figure 7:**
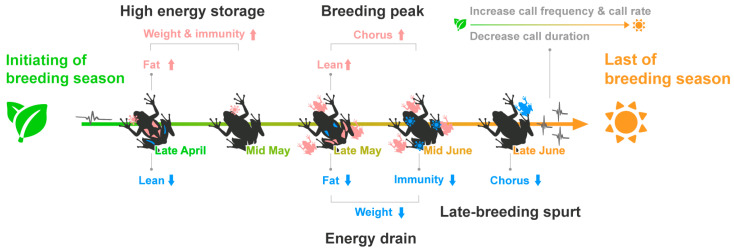
Physiological and behavioral variations of Japanese tree frogs vary with temporal changes in the breeding season. Frogs that emerge early have high energy storage and immunity and tend to conserve energy by reducing the cost of calling. In the middle of the breeding season, male frogs call at high density and reach a breeding peak. However, the male frogs that appear at this time are in relatively poor condition, with low energy storage and immunity. This is likely because many of the individuals that started breeding in the early season remain, rather than new individuals participating in the middle period. Towards the end of the breeding season, new individuals are reintroduced, and male frogs call very rapidly for breeding spurts.

The study aimed to identify physiological and behavioral traits of the Japanese tree frog, which has a prolonged breeding season. The body weight, immunity, and fat content of male frogs were high early in the breeding season but decreased in the middle of the breeding season, suggesting that individuals within the population were maintained for about four weeks. On the other hand, individuals with high fat content and immunity late in the breeding season were likely to be newly introduced individuals. However, the calling pattern did not follow the same pattern as physiology. Early in the breeding season, individuals tended to conserve energy in calling, while later in the breeding season, male frogs tended to have a breeding spurt for mating. Our findings from both physiological and behavioral studies indicate that frogs might alter their involvement in the breeding season depending on their physical and physiological condition (Figure 7). Further research is required to understand how this can be tuned by individual conditions and the environment.

Additionally, breeding peaks with reduced immunity, along with high population density, suggest that prolonged breeder species may be most susceptible to disease during this time. Therefore, it is necessary to be particularly careful about the timing of breeding peaks when related species are threatened by disease. Since breeding in prolonged breeder species is more complicated than in exclusive breeder species, more research is needed. More research into their reproduction remains to be performed as it can provide important information for developing conservation strategies for threatened species, with the study of species conservation and evolutionary strategies.

## Figures and Tables

**Table 1:** Mean ± standard deviation (SD) of temperature and humidity investigated in the field during five observation rounds (R1–R5). Significant differences (*p* < 0.05) were determined using Dunn’s Post Hoc test from Kruskal–Wallis test and are represented by lowercase letters.

Factor	R1	R2	R3	R4	R5
Temperature	17.9 ± 0.51 ^a^	19.87 ± 1.27 ^ab^	20.86 ± 0.67 ^bc^	24.21 ± 0.54 ^cd^	26.27 ± 0.48 ^d^
Humidity	41.67 ± 2.26 ^a^	46.77 ± 5.53 ^ab^	59.49 ± 0.66 ^bc^	72.65 ± 0.83 ^cd^	80.98 ± 0.45 ^d^

**Table 2:** The two major components obtained from chorus size, body condition (length and weight), physiological condition (bacterial killing ability (BKA), fat content, and lean body content), and call parameters (note duration, dominant frequency, and call rate) using principal component analysis (PCA) are presented, along with the loading coefficients for RC1 and RC2 components.

Components	RC1	RC2	Uniqueness
Note duration	0.796		0.349
Frequency	−0.770		0.409
Call rate	−0.707		0.500
Body weight	0.708		0.310
Body length	0.484		0.677
BKA	0.652		0.563
Fat content		0.929	0.118
Lean body content		−0.924	0.106
Testosterone		0.502	0.714
Chorus size			0.853

## Data Availability

Not applicable.

## References

[1] Wells K.D., Schwartz J.J., Wells K.D. (2007). The behavioral ecology of anuran communication. Hearing and Sound Communication in Amphibians.

[2] Hirai T., Matsui M. (2000). Feeding habits of the Japanese tree frog, *Hyla japonica*, in the reproductive season. Zool. Sci..

[3] Bevier C.R. (1997). Utilization of energy substrates during calling activity in tropical frogs. Behav. Ecol. Sociobiol..

[4] Ryan M.J., Tuttle M.D., Taft L.K. (1981). The costs and benefits of frog chorusing behavior. Behav. Ecol. Sociobiol..

[5] Schwartz J.J., Rand A.S. (1991). The consequences for communication of call overlap in the Túngara frog, a neotropical anuran with a frequency-modulated call. Ethology.

[6] Wells K.D., Taigen T.L. (1989). Calling energetics of a neotropical treefrog, *Hyla microcephala*. Behav. Ecol. Sociobiol..

[7] Klump G.M., Gerhardt H.C. (1987). Use of non-arbitrary acoustic criteria in mate choice by female gray tree frogs. Nature.

[8] Park J.-K., Do Y. (2022). Wind Turbine Noise Behaviorally and Physiologically Changes Male Frogs. Biology.

[9] Titon S.C.M., de Assis V.R., Junior B.T., Barsotti A.M.G., Flanagan S.P., Gomes F.R. (2016). Calling rate, corticosterone plasma levels and immunocompetence of *Hypsiboas albopunctatus*. Comp. Biochem. Physiol. Part A Mol. Integr. Physiol..

[10] Braude S., Tang-Martinez Z., Taylor G.T. (1999). Stress, testosterone, and the immunoredistribution hypothesis. Behav. Ecol..

[11] Desprat J.L., Lengagne T., Dumet A., Desouhant E., Mondy N. (2015). Immunocompetence handicap hypothesis in tree frog: Trade-off between sexual signals and immunity?. Behav. Ecol..

[12] Wells K.D., Wells K.D. (2007). Anuran vocal communication. The Ecology and Behavior of Amphibians.

[13] Wells K.D. (1977). The social behaviour of anuran amphibians. Anim. Behav..

[14] Saenz D., Fitzgerald L.A., Baum K.A., Conner R.N. (2006). Abiotic correlates of anuran calling phenology: The importance of rain, temperature, and season. Herpetol. Monogr..

[15] Gastón M.S., Vaira M. (2020). Male mating success is related to body condition and stress-induced leukocyte response in an anuran with scramble competition. Can. J. Zool..

[16] Greene A.E., Funk W.C. (2009). Sexual selection on morphology in an explosive breeding amphibian, the Columbia spotted frog (*Rana luteiventris*). J. Herpetol..

[17] Wheeler C.A., Hartwell H., Welsh J. (2008). Mating strategy and breeding patterns of the foothill yellow-legged frog (*Rana boylii*). Herp. Cons. Biol..

[18] Bevier C.R. (1997). Breeding activity and chorus tenure of two neotropical hylid frogs. Herpetologica.

[19] Murphy C.G. (1994). Determinants of chorus tenure in barking treefrogs (*Hyla gratiosa*). Behav. Ecol. Sociobiol..

[20] Rittenhouse T.A., Semlitsch R.D., Thompson F.R. (2009). Survival costs associated with wood frog breeding migrations: Effects of timber harvest and drought. Ecology.

[21] Rollins-Smith L.A. (2017). Amphibian immunity–stress, disease, and climate change. Dev. Comp. Immunol..

[22] AmphibiaWeb (2006). *Hyla Japonica*; Japanese Tree Frog. https://amphibiaweb.org/species/832.

[23] Hirai T., Matsui M. (2002). Feeding relationships between *Hyla japonica* and *Rana nigromaculata* in rice fields of Japan. J. Herpetol..

[24] Desprat J.L., Mondy N., Lengagne T. (2017). Does testosterone affect foraging behavior in male frogs?. Horm. Behav..

[25] Delgado M., Gutierrez P., Alonso-Bedate M. (1989). Seasonal cycles in testicular activity in the frog, *Rana perezi*. Gen. Comp. Endocrinol..

[26] Madelaire C.B., de Oliveira Cassettari B., Gomes F.R. (2019). Immunomodulation by testosterone and corticosterone in toads: Experimental evidences from transdermal application. Gen. Comp. Endocrinol..

[27] Mendonça M., Chernetsky S., Nester K., Gardner G. (1996). Effects of gonadal sex steroids on sexual behavior in the big brown bat, *Eptesicus fuscus*, upon arousal from hibernation. Horm. Behav..

[28] Assis V.R., Titon S.C.M., Barsotti A.M.G., Spira B., Gomes F.R. (2013). Antimicrobial capacity of plasma from anurans of the Atlantic Forest. S. Am. J. Herpetol..

[29] Park J.-K., Do Y. (2020). Evaluating the physical condition of *Hyla japonica* using radiographic techniques. Sci. Total Environ..

[30] Barreira T.V., Tseh W. (2020). The effects of acute water ingestion on body composition analyses via dual-energy X-ray absorptiometry. Clin. Nutr..

[31] Park J.-K., Kim J.B., Do Y. (2021). Examination of physiological and morphological differences between farm-bred and wild black-apotted pond frogs (*Pelophylax nigromaculatus*). Life.

[32] Love J., Selker R., Marsman M., Jamil T., Dropmann D., Verhagen J., Ly A., Gronau Q.F., Šmíra M., Epskamp S. (2019). JASP: Graphical statistical software for common statistical designs. J. Stat. Softw..

[33] Schlaghecke R., Blüm V. (1978). Seasonal variations in fat body metabolism of the green frog *Rana esculenta* (L.). Experientia.

[34] Tattersall G.J., Ultsch G.R. (2008). Physiological ecology of aquatic overwintering in ranid frogs. Biol. Rev..

[35] Keddy-Hector A.C., Wilczynski W., Ryan M.J. (1992). Call patterns and basilar papilla tuning in cricket frogs. II. Intrapopulation variation and allometry. Brain Behav. Evol..

[36] Wilczynski W., Rand S.A., Ryan M.J. (1999). Female preferences for temporal order of call components in the túngara frog: A Bayesian analysis. Anim. Behav..

[37] Wollerman L. (1998). Stabilizing and directional preferences of female *Hyla ebraccata* for calls differing in static properties. Anim. Behav..

[38] Parris K.M., Velik-Lord M., North J.M. (2009). Frogs call at a higher pitch in traffic noise. Ecol. Soc..

[39] Troïanowski M., Mondy N., Dumet A., Arcanjo C., Lengagne T. (2017). Effects of traffic noise on tree frog stress levels, immunity, and color signaling. Conserv. Biol..

